# Exploring the Pathophysiology of ATP-Dependent Potassium Channels in Insulin Resistance

**DOI:** 10.3390/ijms25074079

**Published:** 2024-04-06

**Authors:** Nidia Samara Rodríguez-Rivera, Diana Barrera-Oviedo

**Affiliations:** Laboratorio de Farmacología y Bioquímica Clínica, Departamento de Farmacología, Facultad de Medicina, Universidad Nacional Autónoma de México, Mexico City 04510, Mexico; diana.barrera@facmed.unam.mx

**Keywords:** ionic channels, ATP-dependent potassium channels, insulin resistance

## Abstract

Ionic channels are present in eucaryotic plasma and intracellular membranes. They coordinate and control several functions. Potassium channels belong to the most diverse family of ionic channels that includes ATP-dependent potassium (KATP) channels in the potassium rectifier channel subfamily. These channels were initially described in heart muscle and then in other tissues such as pancreatic, skeletal muscle, brain, and vascular and non-vascular smooth muscle tissues. In pancreatic beta cells, KATP channels are primarily responsible for maintaining the membrane potential and for depolarization-mediated insulin release, and their decreased density and activity may be related to insulin resistance. KATP channels’ relationship with insulin resistance is beginning to be explored in extra-pancreatic beta tissues like the skeletal muscle, where KATP channels are involved in insulin-dependent glucose recapture and their activation may lead to insulin resistance. In adipose tissues, KATP channels containing Kir6.2 protein subunits could be related to the increase in free fatty acids and insulin resistance; therefore, pathological processes that promote prolonged adipocyte KATP channel inhibition might lead to obesity due to insulin resistance. In the central nervous system, KATP channel activation can regulate peripheric glycemia and lead to brain insulin resistance, an early peripheral alteration that can lead to the development of pathologies such as obesity and Type 2 Diabetes Mellitus (T2DM). In this review, we aim to discuss the characteristics of KATP channels, their relationship with clinical disorders, and their mechanisms and potential associations with peripheral and central insulin resistance.

## 1. Introduction

Ionic channels are found in eucaryotic plasma and intracellular membranes. These channels coordinate and control several functions like neurotransmission, secretion, contraction, growth/proliferation, migration, and regulation of cell volume [1]. Ionic channels allow for the passive transport of inorganic ions, such as Na^+^, K^+^, Ca^2+^, and Cl^−^, through the cell membrane. Ionic channels can be gated and do not always allow ions to freely diffuse across the membrane. Mutations in their subunits’ structure can cause important pathologies, known as channelopathies [2].

K^+^ channels belong to the most diverse families of ionic channels. K^+^ channels are present in excitable and non-excitable cells. They control K^+^ efflux and influx and are highly selective due to a very conserved domain known as the selective filter (SF) [3].

Based on their structure, K^+^ channels can be classified according to the primary amino acid sequence of the surrounding pore [4]:

1. Voltage-gated potassium (Kv) channels: channels with six transmembrane motifs and one pore;
2. Potassium inward rectifier channels (KIRs): channels with two transmembrane motifs and one pore;
3. Weak potassium inward rectifier (K2P) channels: channels with four transmembrane motifs and two pores.

In addition, there are other classes of K^+^ channels: the Slo family and the Ca^2+^-activated SK family (SKCa) [5,6].

In humans, K^+^ channels are encoded by more than 90 genes [7]. Mutations in these genes can produce many disorders. For example, mutations in the voltage-gated K^+^ channel encoded by the *KVLQT1* gene and the calcium-modulated K^+^ channel encoded by the *HERG* gene are responsible for long QT syndrome (LQTS) variants [8]. LQTS is a coronary disorder where a delayed heart repolarization after the heartbeat increases the risk of irregular heartbeats which can lead to palpitations, fainting, or sudden death due to ventricular fibrillation [8]. Mutations in the *KCNJ1* gene encoding the KIR1.1a channel induce neonatal Bartter syndrome which is a dominant renal pathology that causes defects in the thick portion of the ascending Henle loop, characterized by hypokalemia, hypochloremia, and metabolic alkalosis [9].

The ATP-dependent potassium (KATP) channels, which belong to the potassium rectifier channel subfamily, are an important group of K^+^ channels. These channels were initially described in heart muscle [10] and were later found to be also present in other types of tissues such as pancreatic beta, skeletal muscle, brain, and vascular and non-vascular smooth muscle tissues [11]. These channels have an octameric structure [12], consisting of four Kir6.1 or Kir6.2 subunits, encoded by the *KCNJ8* and *KCNJ11* genes, respectively, and four regulatory subunits of the SUR1 or SUR2 sulfonylurea receptor, encoded by the *ABCC8* and *ABCC9* genes, respectively [13,14,15]. Kir6.1 is expressed in brain and smooth vascular muscle tissues [11], while Kir6.2 is expressed in brain, cardiac muscle, skeletal muscle, vascular smooth muscle, and pancreatic beta tissues [16,17]. SUR1 is primarily expressed in the brain and pancreatic beta cells [18]. On the other hand, there are two SUR2 isoforms, SUR2A and SUR2B, which are both produced by ABCC9 splicing and are expressed in cardiac, skeletal, and vascular smooth muscle tissues [11]. Different combinations of Kir/SUR subunits generate channels with different cellular functions, tissue locations, and pharmacological profiles [19,20].

Pancreatic KATP channels are mainly SUR1/Kir6.2 channels. Regarding glucose metabolism, more than 100 mutations in SUR1/Kir6.2 channels have been identified that are associated with channelopathies, for example, the recessive form of persistent hyperinsulinemic hypoglycemia of the child, or infantile hyperinsulinemia, and at least two other persistent types of prenatal hypoglycemia [21,22].

In addition to KATP channels, delayed rectifying K^+^ channels (Kv1.x), calcium-activated K^+^ channels (maxi-K), α-adrenoreceptor-activated K^+^ channels, and G protein-activated K^+^ channels are also present in pancreatic beta cells [23,24,25]. It has been proposed that calcium-activated K^+^ channels have a role in triggering the insulin-releasing signal. This process is activated in the initial phases by calcium released from inositol triphosphate (IP3)-mediated intracellular signaling. Nevertheless, channel inhibition has little effect on the total electrical activity of pancreatic beta cells [25]. It is generally accepted that KATP channels are primarily responsible for maintaining the pancreatic beta cell’s membrane potential and are involved in depolarization-mediated insulin release. However, some studies suggest that there are KATP channels that are not involved in insulin release mechanisms and mainly work on the second phase of the process [26,27,28,29]. In this review, we discuss the pathophysiological features, processes, and possible relationships between KATP channels and insulin resistance in peripheral tissues and the brain.

## 2. ATP-Dependent Potassium Channels (KATPs): Characteristics and Regulation

From a pharmacological perspective, KATP channels can be divided into two groups based on their sensitivity to sulfonylureas. The first group corresponds to the types of neuronal, neuroendocrine, and beta cells that are 100 to 1000 times more sensitive to sulfonylureas than the channels of the second group. The channels of this group contain the SUR2 subunit and are mainly expressed in cardiac and muscular tissues [30]. By using compounds that activate them or increase the channels’ average opening time, such as diazoxide and pinacidil (potassium channel openers: KCOs), it is also possible to distinguish between the compositions of different groups of channels. KATP channels containing SUR1 and SUR2B subunits respond better to diazoxide than those with SUR2A (which are mainly expressed in cardiac tissues) [31,32,33], and it is possible to distinguish between channels constituted by SUR2A or SUR2B due to their differential sensitivity to pinacidil [33,34].

KATP channels can be considered moderate rectifier channels; i.e., they conduct K^+^ more efficiently into the cell than outward [30]. The degree of rectification depends on the presence of cytoplasmic Mg^2+^ or polyamines, such as putrescine and spermidine, which can block the entry of K^+^ when the membrane is depolarized [35].

## 3. KATP Channel Activity and Functional Regulation

In pancreatic beta cells, the KATP channels consist of Kir6.2/SUR1 tetramers [36]. When extracellular glucose is increased, there is a reduction in the KATP opening paired with insulin release [37]. According to these results, KATP channels are key components that relate glucose metabolism, membrane electrical activity, and insulin release [30,37]. They also provide the dominant K^+^ conductance at rest and determine the beta-cell membrane potential [22]. Pancreatic KATP channels are blocked by sulfonylureas and activated by diazoxide [33,38].

**Figure 1:**
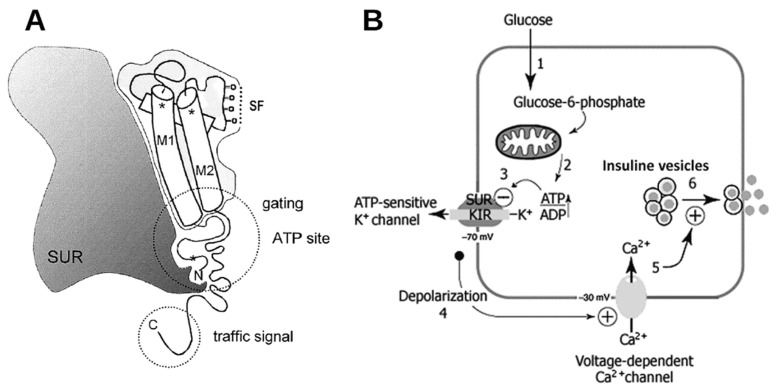
(**A**) Schematic representation of SURx with Kir6.x subunit showing the most studied Kir domains, the pore region (M1 and M2), the potassium sensor region or selective filter (SF), the ATP-binding site, and the carboxyl end where the membrane transport signal is located. (**B**) Schematic representation of pancreatic beta KATP channel signaling. When glucose enters the cell (1), its metabolism increases the ATP concentration (2); ATP blocks KATP channels (3), causing membrane depolarization (4) that leads to the opening of voltage-dependent Ca^2+^ channels (5). The increase in the intracellular Ca^2+^ concentration triggers the release of insulin-containing vesicles (6). SURx: neither SUR1 or SUR2; Kir&.x: neither Kir6.1 or Kir6.2. (Modified from Aguilar-Bryan and Bryan, 1999, Adapted with permission from OXFORD UNIVERSITY PRESS Licence number: 5760270637542. Copyright © 1999, Copyright © 1999 by The Endocrine Society. More details on “Copyright and Licensing” are available via the following link: https://s100.copyright.com/AppDispatchServlet (accessed on 8 January 2024) and Flanagan et al., 2009. Adapted with permission from John Wiley and Sons, Licence number: 5760290001626. Copyright © 2008 Wiley-Liss, Inc. More details on “Copyright and Licensing” are available via the following link: https://s100.copyright.com/AppDispatchServlet (accessed on 8 January 2024)).

When extracellular glucose levels increase, glucose is transported into the cytosol by glucose transporter 2 (GLUT-2), and ATP production increases. Kir6.2 is non-cooperatively blocked by ATP, and the Kir6.2/SUR1 complex’s ATP inhibitory concentration (CI_50_) decreases by an order of magnitude [39]. On the other hand, SUR1 is activated by ADP/Mg^2+^, which binds to the nucleotide-binding domain 2 (NBD) [40]. When the extracellular glucose concentration is less than 3mM, the membrane potential remains between −60 and −70 mV. The increase in cytosolic glucose produces a gradual depolarization which causes KATP channel closure. After KATP channel closure, the membrane depolarizes to −50 mV, triggering an action potential that activates L-type voltage-gated Ca^2+^ channels (L-Ca^2+^) and probably also the type T (T-Ca^2+^) channels. This can cause an influx of Ca^2+^ that triggers insulin/Zn^2+^ transporter granule exocytosis [22,30] (Figure 1).

The electrical activity of KATP channels is complex. The increase in plasma glucose levels leads to a sustained initial depolarization that promotes a slow and sustained increase in the intracellular Ca^2+^ concentration (presumably due to T-Ca^2+^channels) leading to a period of insulin release known as phase 1. Oscillatory changes in the membrane electrical activity coincide with the oscillatory changes in the intracellular Ca^2+^ concentration caused by L-Ca^2+^ channels. These changes lead to pulses of insulin release from the pancreatic islets; this period is known as phase 2 [41,42].

Patch-clamp experiments have shown that, in the absence of ATP, KATP channels remain open and lose their activity [43]. Adenine dinucleotides can inhibit channel activity in Mg^2+^-free solutions [33]. The mechanism of KATP channels’ on/off process is related to the ATP/Mg-ADP ratio [44], and anionic phospholipid hydrolysis stabilizes the structure of the channel into a functional state [45,46,47,48].

The ATP IC_50_ is 5–10 µM for pancreatic beta-cell channels [49], 8–500 µM for cardiac channels [39], and 20–100 µM for SUR2A/Kir6.2 channels [50]. The physiological range of intracellular Mg-ATP concentrations is around 1 mM, which is above the in vitro concentrations needed for the inactivation of KATP channels. Under these conditions, a 99% inhibition of the channel would be predicted, which raises the question of the mechanism that ties metabolism to the in vivo channel conditions. It has been proposed that KATP channels might be regulated by the large fluctuations in intracellular ATP concentrations or the existence of some compartmentalization that allows for local changes in the ATP concentration that are capable of inhibiting KATP channels [51]. Interestingly, pancreatic KATP channels (SUR1/Kir6.2) have a higher IC_50_ with Mg-ATP than with ATP, suggesting that the inhibition potential is related to the magnitude of negative ion charges [52]. However, in cardiac channels, the opposite occurs (SUR2A/Kir6.2 channels), where a greater inhibition of the channel is obtained with ATP in the presence of Mg^2+^ [53]. Yet, when studying SUR1 (*ABCC8*) polymorphisms related to infantile hyperinsulinism, it was found that the regulation of these channels includes mechanisms other than ATP control.

In vitro studies with reconstituted SUR1 channels containing a glycine-to-arginine mutation at the 1479 site (SURG1479R) showed that the channels were inhibited by ATP and Mg-ATP in the same way as wild-type channels, although these mutant channels were not activated by Mg-ADP or diazoxide [54]. This mutation is located in the NBD. Additionally, it is known that ADP and other diphosphate nucleosides inhibit KATP channels in the absence of Mg^2+^, so the presence of Mg^2+^ is critical for its activation [30,55]. KATP channel regulation relies on changes in both ATP and Mg-ADP concentrations [56]. Currently, this regulation is not well understood. Despite these channels having been known for more than three decades, there are few studies on the behavior of KATP channels in response to variations in intracellular ATP/ADP concentrations at a physiological level.

In studies with truncated carboxyl terminal Kir6.2 subunits, and in the absence of SUR1 subunits, it was found that Kir6.2 subunits are sufficient to form the pore channel structure and that these subunits possess the ATP-mediated inhibitory activity of KATP channels [57,58]. However, these channels are not activated by Mg-ADP or other activators such as diazoxide, and they are not inhibited by sulfonylureas [57,58]. On the other hand, these activities were restored when expressing SUR1 along with the mutated Kir6.2 subunits [48,57]. That is, the pore-forming units of the beta-cell KATP channels are the Kir6.2 subunits, which contain the ATP regulation sites. The SUR1 units are the regulatory units that can control the channel activity. The Kir6.2 pore conformation can force the channel into an “off” state in which they are not sensitive to ATP inhibition, potentially via the action of Mg-ADP and activators such as diazoxide in one or both NBD domains of SUR1 [48,59,60].

Another important KATP channel regulator is phosphatidyl inositol 4-5-bisphosphate (PIP2), which blocks the activity of these channels in a nucleotide-independent way [44]. PIP2 stabilizes the open state of the KATP channel without affecting the individual channels’ current amplitude [46,61]. It has been demonstrated that the PIP2 and KATP channel interaction occurs in the cytoplasmic Kir6.2 region; this interaction can be blocked by neomycin or poly-L-lysine and is dependent on the phosphorylation status of the Kir6.2 amino-terminal region [48,58]. Similarly, the interaction between the Kir6.2 and SUR1 subunits depends on the phosphorylation of this region and is also involved in the coupling of sulfonylureas to SUR1 for Kir6.2 pore blockage [62]. PIP2 decreases the channels’ sensitivity to ATP via intracellular mechanisms [63]. It is currently proposed that short-term physiological regulation of KATP channel is mediated by Mg-ATP while long-term regulation depends on PIP2 [61,64].

## 4. Genetic Variability in Human KATP Channels: Insights and Implications in Insulin Resistance

The *KCNJ11* (Kir6.2) and *ABCC8* (SUR1) genes encode for Beta-pancreatic KATP channels and are located on the same human chromosome (11p5.1) with 4.5 kb between them. *KCNJ11* has a unique exon that encodes for a 390-amino-acid protein consisting of two transmembrane regions and the pore-forming subunit [65]. *ABCC8* only has one open reading frame (ORF) with 39 exons and more than 100 kb of genomic DNA and a median of 124 bp per exon.

Polymorphisms have been described for both genes, and some of them are related to pathologies such as hypo- and hyperinsulinemia (HI) of infancy and familiar or PHHI [9,66]; these polymorphisms may also influence the susceptibility to Type 2 Diabetes Mellitus (T2DM) and the channels’ pharmacological response [67,68,69,70,71].

Mutations in *ABCC8* are the most frequent cause of HI. These mutations can be divided into two functional types: class I mutations which reduce the number of channels on the membrane by interrupting channel complex biogenesis and class II mutations which reduce the channel opening rate by preventing Mg-ADP activation or by producing defective proteins incapable of forming pores [22,66,72]. Mutations in *KCNJ11* are less frequent causes of HI and also show low or no channel activity. Sulfonylureas can act as chemical chaperones correcting traffic deficiencies in class I mutations [73], while KCOs like diazoxide are used for treating class II mutations. Nevertheless, gain-of-function mutations keep the channels open and beta cells hyperpolarized, thereby decreasing insulin secretion [74].

## 5. KATP Channels’ Role in Insulin Resistance in Peripheral Tissues

In vitro studies suggested that sulfonylureas have a mimetic insulin effect in extra-pancreatic tissues by improving glucose recapture through an insulin-independent pathway [75,76] or by potentiating insulin action [77,78]. This effect might be due to the SUR subunits’ sensitivity to sulfonylureas. For example, skeletal muscle KATP channels (mainly Kir6.2/SUR2A) are between 30 and 300 times less sensitive to glibenclamide in comparison to pancreatic beta KATP channels [79].

An increase in glucose oxidation and ATP production was observed in the hearts of rats that were perfused with tolbutamide. An increase in glucose recapture was also observed in rat skeletal muscle [80,81]. On the other hand, a concentration- and time-dependent increase in glucose recapture was observed in the L6 skeletal muscle cell line treated with glyburide and gliclazide. Additionally, glucose-independent GLUT1 translocation to the cellular membrane has also been observed, but in an insulin-dependent way, with no increase in GLUT-3 and GLUT-4 translocation [78,82]. The same effect was observed for gliclazide in rat skeletal muscle ex vivo experiments, although the increase in glucose recapture was abolished by the blockage of KATP channels with diazoxide [83].

Kir6.2-knockout mice have no KATP channel activity in pancreatic beta, skeletal muscle, and cardiac cells. In general, these mice had a closed KATP channel phenotype where the pancreatic beta cell’s membrane was constitutively depolarized. In addition, at a concentration of glucose less than 3mM, significantly high concentrations of Ca^2+^ and levels of insulin secretion were achieved. This phenotype could not be restored by KCOs. In general, compared to wild-type mice, these mice presented a lower glucose intolerance and higher insulin sensitivity [84].

SUR1-knockout mice’s beta cells presented a similar electrophysiological phenotype to Kir6.2-knockout beta cells, but the mice maintained normal glycemic rates and presented an increase in insulin sensitivity [85].

On the other hand, in a study that analyzed different *KCNJ11* polymorphisms in a Danish population, none of the studied polymorphisms affected insulin release from beta cells after glucose or tolbutamide administration, but there was a significant increase (62%) in insulin sensitivity in comparison to subjects that did not have the *KCNJ11* polymorphisms [86]. It is possible that KATP channels are involved in insulin-dependent glucose recapture in skeletal muscle. It has been shown that KATP channel inhibition in peripheral tissues, mainly in skeletal muscle, improves insulin sensitivity [87].

Nicorandil, a KCO used as a human angina treatment, decreases glucose metabolism, causing insulin resistance without interfering with insulin secretion [87]. In other in vitro studies with human skeletal muscle cells, nicorandil and PCO-400 (another KCO) inhibited insulin-mediated glucose recapture in a dose-dependent manner. This inhibition was reverted by gliclazide or glibenclamide pre-treatment, also in a dose-dependent manner [86]. In the rat aorta, phorbol 12-myristate 13-acetate (PMA), a PKC activator, was able to revert PCO-400 glucose recapture inhibition in a dose-dependent manner [33,79]; however, other works have reported no change in PKC levels after PCO-400 or PMA stimulation [88].

**Figure 2:**
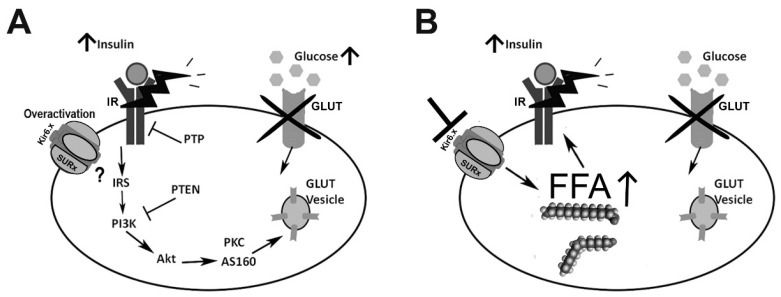
KATP channels and insulin resistance in peripheral tissues. (**A**) In skeletal and muscle tissue, KATP channel activation may be related to insulin resistance and a decrease in glucose recapture under high insulin and glucose conditions. The molecular mechanisms involved have yet to be elucidated. (**B**) In contrast, in adipocytes, sustained KATP channel inhibition promotes fatty acid synthesis which may lead to obesity and insulin resistance. KATP: ATP-Dependent Potassium.

These results suggested that KATP channel opening decreases glucose recapture in target tissues in an insulin- and high-glucose-concentration-dependent manner; therefore, KATP channel opening can potentially contribute to insulin resistance in vivo (Figure 2A).

Besides KATP channels’ effects on glucose metabolism, they have been shown to have effects on lipid metabolism. SUR1 is expressed in human adipocytes where it regulates intracellular Ca^2+^ concentrations [89]. In 3T3-Li adipocytes, diazoxide inhibits the insulin-mediated activation of the fatty acid synthase (FAS) [90]. Glibenclamide stimulates FAS activity, increases glycerol 3-phosphate dehydrogenase activity, and inhibits lipolysis [91].

These works suggest that the opening of KATP channels has an inhibitory effect on fatty acid synthesis. This can be related to changes in intracellular Ca^2+^ concentrations which are a known regulator of lipogenesis, lipolysis, and triglyceride synthesis [91]. As previously mentioned, the increase in free fatty acids (FFAs) might cause insulin resistance; therefore, pathological processes that lead to prolonged adipocyte KATP channel inhibition might promote obesity due to insulin resistance (Figure 2B).

It has been shown that there is a strong correlation between intracellular triglyceride levels and insulin resistance outcomes (i.e., in skeletal muscle and hepatic tissues) [92,93]. Some studies suggested that esterified FFAs can activate KATP channels in skeletal and cardiac muscle [94,95,96]. Esterified FFAs, such as oleoyl-CoA, can activate pancreatic beta KATP channels at low concentrations (1 µM). They can also block ATP inhibition. This activation occurs through the interaction of Kir6.2 with Mg-ADP or other KCOs, but not with SUR1 [33]. This suggests that KATP channels containing Kir6.2 in other tissues, such as smooth or skeletal muscle, can similarly react to fatty acids. An increase in the intracellular esterified fatty acid concentration due to long-term exposure to FFAs could increase KATP channel activity and, as a consequence, cause insulin resistance [96].

## 6. Beyond the Blood–Brain Barrier: Understanding the Effects of Insulin Resistance on the Brain

The brain has different mechanisms for detecting and regulating glucose metabolism. It receives natural impulses from peripheral tissues, but it also has neurons that can determine glycemic levels by altering their firing pattern. When the glucose level increases, glucose-responsive (GR) neurons increase their firing pattern, and glucose-sensitive (GS) neurons reduce their firing [97]. These neurons are located in the lateral hypothalamic area (LH), ventral medial hypothalamic area (VMH), nucleus tractus solitarius, and other areas [98,99,100].

It has been proposed that GR neurons use KATP channels to regulate their firing rate [101]. Brain KATP channels contain Kir6.2 and either SUR1 or SUR2 as the regulatory subunits [11,49,101]. As in pancreatic beta cells, neural KATP channels are inhibited by ATP, causing membrane depolarization and triggering action potentials or firing [102]. Nevertheless, in basal conditions, the levels of interstitial glucose are lower than peripheral levels (<2 mM), and changes in the extracellular concentrations are relatively small (<1 mM). It is not clear how such small changes can alter the ATP/ADP ratio enough to regulate KATP channels. Additionally, Kir6.2 and SUR1 expression is not limited to GR neuron regions [101].

**Figure 3:**
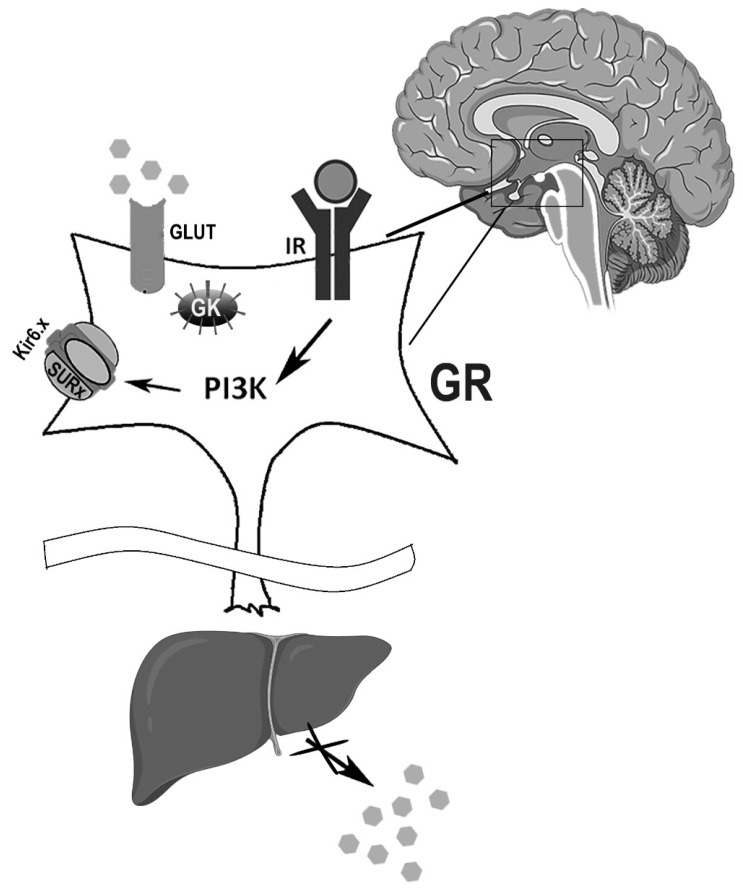
Insulin-mediated KATP effects on the brain. In glucose-responsive (GR) neurons, KATP channels regulate the neurons’ firing rate, and insulin regulates the KATP channel electrical activity. KATP channels act as glucose sensors in glucose transporter (GLUT)- or glucokinase (GK)-containing neurons, preventing insulin resistance and glucose intolerance. At the peripheral level, brain insulin regulation of KATP activity may prevent an increase in hepatic glucose production and protect against insulin resistance. IR: insulin receptor; PI3K: phosphatidylinositol 3-kinase. Gray hexagons represent glucose molecules.

In pancreatic beta cells, glucokinase (GK) is a regulator that controls ATP levels produced by glycolysis, and it is also expressed in the brain [21,103]. Levin et al. [97] have proposed that KATP channels work as glucose sensors in the neurons that contain a glucose transporter and/or GK, or any other hexokinase located sufficiently close to the plasma membrane and to KATP channels (Figure 3) [97].

Insulin is not the only glucose metabolism regulator in the brain [104]. Insulin receptors (IRs) are abundantly expressed in all areas of the brain [105,106]. Insulin’s interaction with IRs activates phosphatidylinositol-3 kinase (PI3K), which regulates the electrical activity of the target neurons through KATP channel stimulation (Figure 3) [107]. The channel activation induces hyperpolarization and a reduction in the activity of the target neuron [108]. Brain IR-knockout mice are overweight, insulin-resistant, and glucose-intolerant [109]. It has also been observed that hypothalamic IR chronic blockade leads to pancreatic insulin resistance and to an increase in hepatic glucose production (Figure 3) [110,111].

Infusion of insulin or insulin-mimetic peptides in the third ventricle suppresses liver glucose production, while central suppression of insulin signaling decreases the ability of circulating glucose to inhibit glucose production. This effect of insulin on neurons was shown to be mediated by neural KATP channel activation which can be inhibited by tolbutamide [111].

Hypothalamic KATP channels are activated by insulin and can influence hepatic glucose production by diminishing the effects of 6P-glucose and phosphoenol pyruvate carboxylase that are mediated by the autonomous nervous system [112]. It is not clear yet how the environment and genetic background can affect these neuronal pathways.

Finally, another insulin resistance type has been identified at the central level in obese subjects and individuals with the G972R polymorphism in *IRS-1*, which is associated with T2DM and peripheral insulin resistance [84]. It is possible that brain insulin resistance could be the initial trigger for the development of T2DM and obesity, rather than [113].

## 7. Conclusions

KATP channels play a very important role in energetic regulation, not only in pancreatic beta cells but also in all insulin target tissues. Alterations in the correct functioning of these channels lead to pathologies such as diabetes and obesity, both of which have a metabolic outset and can originate from an increase in peripheral and central insulin resistance.

There are diverse mechanisms that lead to insulin resistance, one of which involves KATP channels and their control of the insulin pathway in different tissues.

In peripheral tissues, the activation of extra-pancreatic KATP channels decreases membrane glucose receptor expression and glucose recapture and leads to RI. The opposite occurs when these channels are inhibited. In lipidic tissue, the inhibition of KATP channels promotes fatty acid synthesis, and their activation promotes lipolysis. High fatty acid levels lead to insulin resistance in fat and other tissues. An increase in circulating free fatty acids also causes an increase in intracellular esterified fatty acids, increasing their interaction and the activation of KATP channels leading to insulin resistance.

In central tissues, KATP channels are activated by insulin, and the disruption of this pathway (by IR or PI3K inhibition) leads to peripheral or central insulin resistance; therefore, KATP channel activation protects against insulin resistance, while KATP channel inhibition promotes it.

Gain- and loss-of-function mutations in KATP channels have been associated with pathologies like hyperinsulinemia and different types of diabetes. The first manifestations of these pathologies, such as IR, are not commonly studied. It would be interesting to investigate whether there is a genetic background in the KATP channel genes that could help us to more precisely explain these channels’ role in insulin resistance.

For example, mutations that lead to an increase in the open probability of extra-pancreatic channels at the Kir6.2 level could represent an important component for predisposition to insulin resistance. Meanwhile, mutations leading to a minor open probability can have a protector effect in peripheral tissues, with the opposite effect expected in central tissues. Thus, it is important to view KATP channel research from this perspective, such as in the case of obesity and T2DM, in order to discover better prevention tools rather than treatments for these pathologies.
